# Linking pollen foraging of megachilid bees to their nest bacterial microbiota
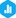


**DOI:** 10.1002/ece3.5599

**Published:** 2019-09-02

**Authors:** Anna Voulgari‐Kokota, Markus J. Ankenbrand, Gudrun Grimmer, Ingolf Steffan‐Dewenter, Alexander Keller

**Affiliations:** ^1^ Department of Bioinformatics Biocenter University of Wuerzburg Wuerzburg Germany; ^2^ Center for Computational and Theoretical Biology University of Wuerzburg Wuerzburg Germany; ^3^ Department of Animal Ecology and Tropical Biology Biocenter University of Wuerzburg Wuerzburg Germany

**Keywords:** foraging patterns, nest microbiota, plant–microbe–pollinator triangle, pollination network, solitary bees, wild bees

## Abstract

Solitary bees build their nests by modifying the interior of natural cavities, and they provision them with food by importing collected pollen. As a result, the microbiota of the solitary bee nests may be highly dependent on introduced materials. In order to investigate how the collected pollen is associated with the nest microbiota, we used metabarcoding of the ITS2 rDNA and the 16S rDNA to simultaneously characterize the pollen composition and the bacterial communities of 100 solitary bee nest chambers belonging to seven megachilid species. We found a weak correlation between bacterial and pollen alpha diversity and significant associations between the composition of pollen and that of the nest microbiota, contributing to the understanding of the link between foraging and bacteria acquisition for solitary bees. Since solitary bees cannot establish bacterial transmission routes through eusociality, this link could be essential for obtaining bacterial symbionts for this group of valuable pollinators.

**Open Research Badges:**

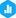
This article has earned an Open Data Badge for making publicly available the digitally‐shareable data necessary to reproduce the reported results. The data is available at https://www.ebi.ac.uk/ena/data/view/PRJEB27223, https://www.ebi.ac.uk/ena/data/view/PRJEB31610, and https://doi.org/10.5061/dryad.qk36k8q

## Graphical abstract

We investigated the importance of imported pollen for the solitary bee nest microbiomes, as its inclusion introduces a wide diversity of environmental and primarily floral bacteria. The simultaneous investigation of pollen composition and nest microbiota showed that both bacterial diversity and composition can be influenced by the foraging preferences of each bee species. Also, we were able to suggest associations with specific bacterial taxa as possibly secured through plant visitation. The present study contributes to the understanding of the complex relationships in the plant–pollinator–microbe triangle.

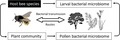

## 1 INTRODUCTION

The bee gut microbiota plays an important role in bee metabolism, immune function, and physiological development (Engel, Martinson, & Moran, 2012). In social beehives, the nursing of the offspring does not only offer protection and food provision, but it also secures transmission of symbiotic bacteria, which aid in nutrition and pathogen defense (Anderson, Sheehan, Eckholm, Mott, & DeGrandi‐Hoffman, 2011; Vásquez et al., 2012; Vásquez & Olofsson, 2009). More specifically, the larval life cycle of the well‐studied honeybee takes place in the antimicrobial environment of the hive with bacterial taxa such as Lactobacilli and Acetobacteriaceae being transferred to the larvae through active nursing by nest mates (Kwong & Moran, 2016; Martinson, Moy, & Moran, 2012; Vojvodic, Rehan, & Anderson, 2013). The benefits of a hive system also include direct acquisition of essential bacterial symbionts through social interaction between individuals of the same or different generations (Martinson et al., 2012). The worker honeybee gut microbiota during the adult life in the hive is characterized by less than ten core bacterial taxa, which have been functionally characterized (Kwong & Moran, 2016; Moran, Hansen, Powell, & Sabree, 2012; Powell, Martinson, Urban‐Mead, & Moran, 2014).

On the other hand, solitary bee nests harbor highly diverse bacterial communities (Keller, Grimmer, & Steffan‐Dewenter, 2013; Lozo et al., 2015; McFrederick & Rehan, 2016; Mohr & Tebbe, 2006; Voulgari‐Kokota, Grimmer, Steffan‐Dewenter, & Keller, 2019). However, only few studies have dealt with their role in larval health (Keller et al., 2018; McFrederick, Vuong, & Rothman, 2018) or their acquisition routes (McFrederick et al., 2017; Voulgari‐Kokota, McFrederick, Steffan‐Dewenter, & Keller, 2019). Bees leading a solitary lifestyle with no direct contact between multiple generations are excluded from the benefits of a social structure, where individuals are in constant interaction with their nestmates, and their nest microbiota is more susceptible to environmentally introduced bacteria (Keller et al., 2013; McFrederick et al., 2017; Rothman, Andrikopoulos, Cox‐Foster, & McFrederick, 2018; Voulgari‐Kokota, Grimmer, et al., 2019; Voulgari‐Kokota, McFrederick, et al., 2019). As a result, the establishment of steady host–microbe interactions requires different mechanisms and could be the result of more complex processes, where beneficial microbial interactions are reinforced and negative interactions with pathogens are minimized. These should be able to secure transmission routes over multiple generations through active and passive transmission. Three possible mechanisms are active inoculation of nest structures (as in Kaltenpoth, Yildirim, Gürbüz, Herzner, & Strohm, 2012) including stored pollen, active inoculation of eggs (Hosokawa, Kikuchi, & Fukatsu, 2007), or passive transmission through imported materials and especially food sources (McFrederick & Rehan, 2016; McFrederick et al., 2017; Rothman et al., 2018).

In the present study, we focused on the importance of the imported pollen in the nest environment, as a major source of introducing environmental and foremost floral bacteria in the solitary bee nest. Solitary bees can be polylectic or oligolectic, and their foraging patterns are determined by the period of their flight activity, their morphological traits, the plant availability, and chemical properties of the plants (Michener, 2007; Westrich, 2015). Considering that the multiple aspects of solitary bee ecology can form distinctive nesting conditions for different species, we test the hypothesis that different pollen foraging patterns could establish different routes for bacterial colonization in the nest. Previous studies have undertaken the task of characterizing solitary bee nest microbiota (Keller et al., 2013; Lozo et al., 2015; McFrederick et al., 2017; Voulgari‐Kokota, Grimmer, et al., 2019) or have identified the plant composition of pollen provisions (Danner, Keller, Härtel, & Steffan‐ Dewenter, 2017; Sickel et al., 2015; Villanueva‐Gutiérrez & Roubik, 2016). Simultaneous examination of pollen composition and pollen microbiota from the nests of a wild bee species has shown covariation across different landscapes, even though the causality of this association was not clear (McFrederick & Rehan, 2018). Furthermore, the association between the pollen microbiota and the pollen composition in the nests of a single bee species suggested possible plant‐mediated host–microbe relationships (McFrederick & Rehan, 2016). However, the complex relationships in the plant–pollinator–microbe triangle are currently not well understood and multispecies studies are currently lacking.

In order to investigate the association between pollen types and nest microbiota, we conducted a simultaneous pollen and bacterial metabarcoding survey. We used metabarcoding of the ITS2 rDNA to identify plant species which constituted the pollen provisions in 100 nest chambers coming from seven megachilid bee species, and at the same time, we used metabarcoding of the 16S rDNA to separately characterize the bacterial communities of the same pollen provisions and the respective larvae. Our aim was to identify the degree to which introduced pollen bacteria shape the solitary bee nest microbiota, by testing the hypothesis that the plant diversity and composition of pollen provisions influence the bacterial community of pollen in the bee nests, both at the interspecific and at the intraspecific level, and finally that of the respective larvae. Moreover, we investigated the probability of certain plant species serving as reservoirs of specific bacterial taxa, facilitating conserved bee–microbe associations which result from conserved foraging preferences.

## 2 MATERIALS AND METHODS

### 2.1 Sampling design

Sampling of the solitary bee nests was conducted with the use of 24 artificial trap nests placed at 10 localities throughout an area with an extent of 30 × 25 km in northern Bavaria, Germany. The sampling sites were shaped by agricultural land use and interspersed seminatural vegetation. The trap nests consisted of 30–50 reed internodes (length = 20 cm, width = 4–10 mm) and were placed in early Spring 2016. They were examined every 2 weeks from May until September 2016, and occupied reed canes with clogged entrances were directly transferred into the laboratory. Nests were opened horizontally, and larvae, pollen clumps, and nest material were separately removed with the use of sterile spatula, transferred into sterile tubes, and directly frozen down to −25*°*C. Larvae and respective pollen provisions were selected for this study under the condition that they were not visibly affected by pathogens or parasites and that the larvae had not yet completely consumed the pollen provision. In total, 100 larvae and 100 pollen provisions were included in this study. Of the 100 nest chambers included, 35 belonged to *Heriades truncorum*, 8 to *Megachile ligniseca*, 20 to *Megachile rotundata*, 4 to *Megachile versicolor*, 21 to *Osmia bicornis*, 8 to *Osmia caerulescens*, and 4 to *Osmia leaiana*. The *O. bicornis*, *O. caerulescens*, *M. versicolor*, and 15 of the *M. rotundata* chambers were included in a previous 16S rDNA metabarcoding survey (Voulgari‐Kokota, Grimmer, et al., 2019). The prerequisite for sample inclusion in the present study was the sufficient pollen quantity (>10 mg) in the nest chamber at the time of the nest opening for both bacterial and pollen metabarcoding. All specimens were treated exactly the same way through the whole laboratory workflow, as described in the following chapter.

### 2.2 Laboratory workflow

Genomic DNA isolation from each specimen was conducted with the Macherey‐Nagel NucleoSpin kits for soil (Burbach, Seifert, Pieper, & Camarinha‐Silva, 2016) and food. As a first step, whole larvae were washed with PBS/EDTA solution. The protocol was modified in order to include an extra step of incubation with proteinase K, to ensure lysis of thick bacterial cell walls. After the whole genomic DNA extraction, we proceeded to the PCR amplification of the 16S rDNA for all larvae and pollen sample and the PCR amplification of the ITS2 rDNA for all pollen samples.

We followed the dual‐indexing strategy introduced by Kozich, Westcott, Baxter, Highlander, and Schloss (2013) in order to generate a pooled amplicon library based on the 16S rRNA V4 variable region for the Illumina platform (Illumina, 2017). Primers used to amplify the V4 region were as follows: AATGATACGGCGACCACCGAGATC TACAC [8bp ‐i5 index] ATGGTAATTGTGT‐ GCCAGCMGCCGCGGTAA and CAAGCA GAAGACGGCATACGAGAT [8bp ‐i7 index] AGTCAGTCAGCCGGACTACHVGGGTWTCTAAT (Illumina, 2016a). The same dual index strategy was used to generate the pooled amplicon library for the ITS2 rDNA region used for pollen metabarcoding. We used a combination of plant barcoding primers expanded for Illumina conformity, as described in Sickel et al. (2015). The primer sequences were as follows: AATGATACGGCGACCACCGAGATCTACAC [8bp ‐i5 index] CCTGGTGCTGGTATGCGATACTTGGTGTGAAT and CAAGCAGAAGACGGCAT‐ ACGAGAT [8bp ‐i7 index] AGTCAGTCAGCCTCCTCCGCTTATTGATATGC‐3. The primers amplify a total fragment of approximately 470–480 bp, including the complete ITS2 sequence, enabling safe plant identification up to species level.

PCRs were conducted in triplicates with 1 µl of template DNA in each reaction. New England Biolabs (UK) PCR Phusion Master Mix, along with the two indexed primers in a unique combination for each sample and an appropriate quantity of PCR grade dH2O, was used for every reaction. PCR conditions were adjusted according to the primers guidelines. For the 16S rDNA, samples were initially denatured at 95°C for 2 min and then amplified by using 30 cycles of 95°C for 20 s, 55°C for 15 s, and 72°C for 5 min. A final extension (72°C) of 10 min ensured complete amplification. For the ITS2 rDNA, samples were initially denatured at 95°C for 4 min and then amplified with 37 cycles of 95°C for 40 s, 49°C for 40 s, and 72°C for 5 min. For final extension, the program ended with a step of 72°C for 10 min. After the end of the reaction, triplicates were combined and PCR success was checked through gel electrophoresis in a 1% agarose gel.

The V4 16S rDNA library and the ITS2 rDNA library were pooled separately after DNA normalization between samples with the use of the Invitrogen SequalPrep Plate Normalization Kit (Thermo Fisher Scientific, Life Technologies). We used the Bioanalyzer 2200 (Agilent) with High Sensitivity DNA Chips for verification of fragment length distributions. The final pools were also quantified using a Qubit II Fluorometer and the dsDNA High‐Sensitivity Assay Kit (Thermo Fisher Scientific, Life Technologies). The pooled libraries were loaded into 500 cycle reagent Illumina MiSeq cartridges along with the respective read 1 and read 2 sequencing primers. Since the MiSeq requires base diversity on every cycle, libraries were loaded with 5% PhiXv3, a control library for Illumina sequencing runs (Illumina, 2016b). All samples were sequenced in‐house on a MiSeq platform in the Department of Human Genetics of the University of Wuerzburg, Germany.

### 2.3 Data analysis

After data from the 16S rDNA library were acquired, we used fastq‐join v1.3.1 (Aronesty, 2013) to join paired ends of forward and reverse reads. Paired joined reads were accepted if longer than 250bp. We used USEARCH v10.02.240 (Edgar, 2013, 2016) for length truncating, quality filtering, and file conversion. Chimera filtering, operational taxonomic unit (OTU) clustering to a minimum identity of 97%, and OTU table construction were performed also with USEARCH (Edgar, 2013, 2016). We filtered low‐quality reads after setting the maximum number of expected errors at *E*_max_ = 1 (Edgar & Flyvbjerg, 2015). Reads with ambiguous characters or singletons were excluded from the downstream analyses. In the case of the acquired ITS2 rDNA dataset, we kept only the forward reads for downstream analysis, as reverse reads showed less satisfying quality. We filtered reads with high expected error rate or ambiguous characters following the same parameters as described above for the bacterial dataset, and trimmed low‐quality bases at the read ends (<Q30). Reads were accepted if longer than 150 bases. We assigned taxonomy for the de novo picked OTUs of the 16S rDNA library using the RDP v16 reference database up to genus level. The ITS2 rDNA reads were directly mapped against a Bavarian floral reference database for ITS2 (Keller et al., 2015) derived from the ITS2‐database (Ankenbrand, Keller, Wolf, Schultz, & Förster, 2015) with VSEARCH v2.8.4 (Rognes, Flouri, Nichols, Quince, & Mahé, 2016) using an identity cutoff threshold of 97% and global alignments.

Data were further analyzed in R 3.2.4. (R Core Team, 2017) with the packages phyloseq v1.22.3 (McMurdie & Holmes, 2013), vegan v2.5‐2 (Oksanen et al., 2013), and Hmisc v4.1‐1 (Harrell, 2017). The bacterial OTU table was filtered to exclude OTUs annotated as chloroplasts or mitochondria. All samples from both bacterial OTU and plant species datasets were checked to confirm they have more than 1,000 reads after filtering. Wilcoxon tests were conducted to compare the means of bacterial OTU alpha diversity of pollen and larvae for each nest chamber. Furthermore, Spearman's correlations were conducted between the number of identified plant species and the Shannon diversity of bacterial OTUs for all nest chambers.

Beta diversity was visualized with nonmetric multidimensional scaling (NMDS) ordination. The bacterial community distances between samples were based on Bray–Curtis matrices. The distance matrices for pollen composition were based on presence/absence data, since ITS2 rDNA data are not ideal for quantitative community analysis (Bell et al., 2016). PERMANOVA/Adonis was used to test the pollen composition homogeneity between group levels, by subsequently setting the bee species, the sampling site, and the sampling period as independent factors. Pollen and bacterial OTU distance matrices were compared with Mantel tests based on Pearson's product correlation to explore the degree of association between them (Legengre & Legendre, 2012). Ordination and diversity graphs were constructed with ggplot2 v3.0.0 (Wickham, 2009) and reshape2 v1.4.3 (Wickham, 2007).

Descriptions of bacterial and plant communities related to each host species were based on the bacterial OTU taxonomic assignments and identified plant species, respectively. Revealed bacterial and plant communities for each bee species were visualized as bipartite networks after low abundance filtering (>1%) with the package bipartite v2.11 (Dormann, Fruend, & Gruber, 2009). Furthermore, we investigated co‐occurrence patterns between bacterial and plant taxa with the use of a probabilistic approach with the package cooccur v1.3 (Griffith, Veech, & Marsh, 2016), which uses the observed frequencies of co‐occurrence between each pair of taxa and the distribution of each taxon to calculate the respective expected frequencies and return the probabilities that a more extreme value of co‐occurrence could have been obtained by chance. The algorithm defines the observed frequency of co‐occurrence as positive, negative, or random association (Veech, 2013). The analysis was based on matrices of plant species and bacterial taxa agglomerated up to genus level, containing binary data to indicate the absence or presence of each taxon in each sample over a relative abundance threshold of 1%.

Samples were assigned to seven groups according to the pollen composition of their respective provision with the package GMD v0.3.3 (Zhao & Sandelin, 2012), to form groups with samples showing similar foraging patterns. Classification for clustering the plant species into groups was based on generalized minimum distance functions using *k*‐means after cluster number selection according to the Elbow method which takes into account the percentage of variance explained by the clusters against the number of clusters (Kodinariya & Makwana, 2013). We used FENNEC v1.0.5 (Ankenbrand, Hohlfeld, Weber, Förster, & Keller, 2018) to add plant trait information to our pollen composition data and investigate possible phenological differences between the formed plant clusters. Subsequently, we used random forest analysis to assign bacterial communities of pollen and larvae (a) to host bee species and (b) to the defined pollen composition clusters and estimate the significance of these variables for correct classification (Junker & Keller, 2015; Prasad, Iverson, & Liaw, 2006) with the packages varSelRF v0.7‐8 (Diaz‐Uriarte, 2007) and randomForest v4.6‐14 (Liaw & Wiener, 2002). The produced confusion matrices from the random forest analysis include the number of correctly assigned communities to either species or plant clusters as well as class error and total out‐of‐basket (OOB) error rate. To identify indicator bacterial OTUs per tested group, we used variable selection with the OOB error rate estimate set as a minimization criterion.

## 3 RESULTS

We examined 100 megachilid bee nest chambers, which belong to seven solitary bee species. Sequencing for the 16S rDNA generated on average 8,173 high‐quality reads (range from 1,006 to 53,729, *SD* = 8,644.126), while sequencing for the ITS2 rDNA generated on average 13,791.22 high‐quality reads (range from 2,310 to 61,846, *SD* = 5,298.317), after quality and control filtering. For ITS2 reads, 1,958,852 out of 2,139,677 acquired sequences (91.55%) were successfully mapped to the reference database with an identity threshold of >97%. We found clusters for 2,874 bacterial OTUs and assignments for 415 plant species (Appendix S1).

### 3.1 Bacterial communities in larvae and pollen

**Figure 1:**
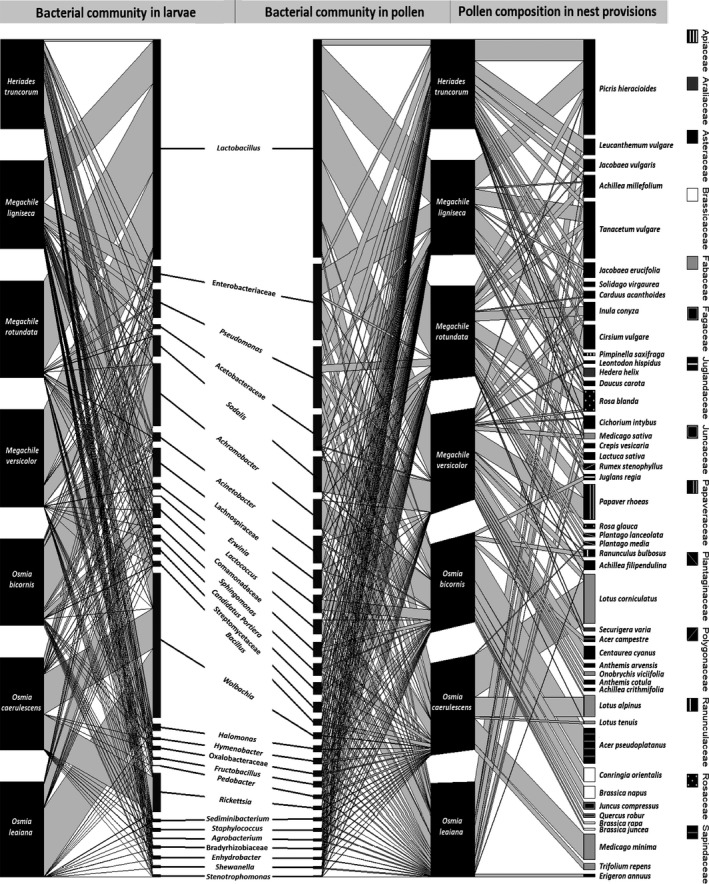
Multilevel networks for interactions between megachilid bee species and plant species as well as between bee species and bacterial taxa (assignment of OTUs up to genus level). Plant species and bacterial taxa are included if they occur in relative abundance of at least 1% in the respective dataset. *Heriades truncorum* is represented with 35 nest chambers, *Megachile ligniseca* with 8, *Megachile rotundata* with 20, *Megachile versicolor* with 4, *Osmia bicornis* with 21, *Osmia caerulescens* with 8, and *Osmia leaiana* with 4 nest chambers

*Lactobacillus* was the most abundant taxon in larvae and pollen of all three *Megachile* species included in this study, while it occurred in all sample groups (Figure 1). The most abundant *Lactobacillus* OTU phylotype was taxonomically assigned as *Lactobacillus micheneri* (100% identity, 230 bp). The phylotype was also phylogenetically close to other wild bee‐associated bacteria such as *Lactobacillus timberlakei* (98.26% identity) and *Lactobacillus quenuiae* (98.26% identity; McFrederick et al., 2018), as well as to the fructophilic *Lactobacillus kosoi* (98.71%; Chiou et al., 2018) and to the honeybee‐associated *Lactobacillus apinorum* (95.22% identity; Olofsson, Alsterfjord, Nilson, Butler, & Vásquez, 2014). *Fructobacillus* is also a genus containing lactic acid bacteria occurring in lower relative abundances in *Megachile* genera in our samples, while *Lactococcus* was the most abundant lactic acid bacterial genus in *O. leaiana* pollen provisions.

Gammaproteobacteria consisted mostly of *Erwinia*, *Pseudomonas*, *Acinetobacter*, and *Halomonas* and are represented in high relative abundance in all sample groups, while they are more prevalent in pollen than in larval bacterial communities (Figure 1). The genera *Rickettsia* and *Achromobacter* are highly abundant in *O. caerulescens* and *O. bicornis* larvae, respectively. The family of Acetobacteraceae, another group belonging to Proteobacteria, is mostly found in pollen provisions of *H. truncorum*, *M. ligniseca*, and *M. rotundata*.

### 3.2 Plant species composition in pollen provisions

The interaction network between bee species and plant species is illustrated in Figure 1. In the nests of three oligolectic bee species, pollen belonged mainly to one plant family. Asteraceae pollen was dominant in *H. truncorum* (91.14%) and *O. leaiana* (98.27%), while pollen provisions from *O. caerulescens* nests were almost entirely composed by Fabaceae (97.24%). The pollen provisions of the other four bee species consisted of more than one plant family.

### 3.3 Co‐occurrence of bacterial taxa with plant species

**Figure 2:**
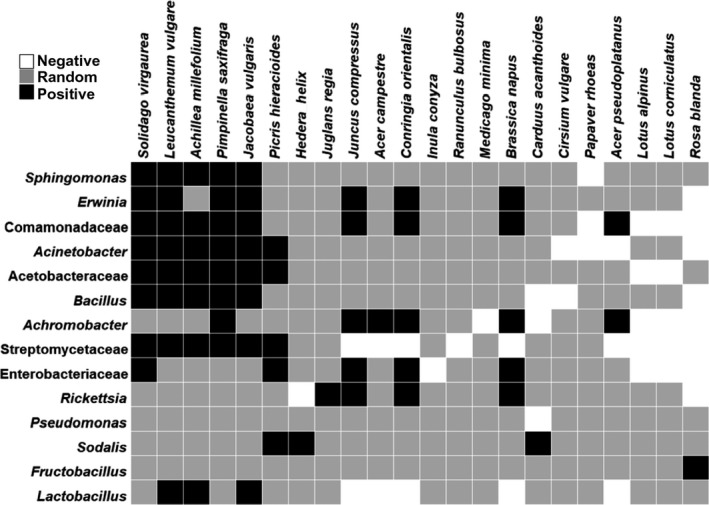
Probabilistic co‐occurrence analysis results for the most abundant bacterial taxa found in pollen and the plant species in pollen provisions. Bacterial taxa shown here are agglomerated up to genus level or up to family level if not better classifiable. Analysis is based on absence–presence data. The relative abundance threshold for a taxon to be considered as existent in a sample is 1%. Taxa showing only random interactions were removed

The probabilistic co‐occurrence analysis investigated which plant species were significantly associated with specific bacterial taxa in the pollen provisions of all nests. Results showed possible connections between bacterial taxa and plant species, marked as positive, negative, and random interactions (Figure 2).

### 3.4 Alpha diversity of bacterial OTUs and pollen plant species

**Figure 3:**
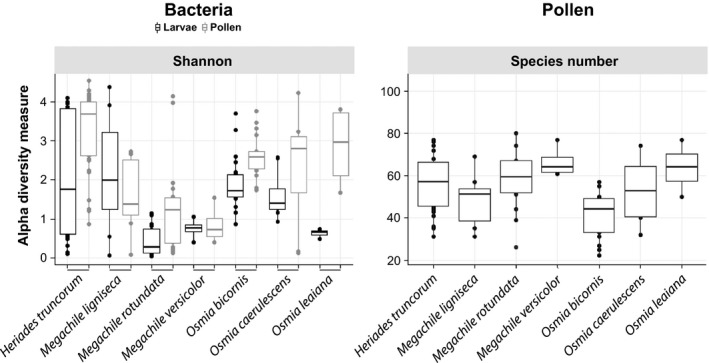
Left: Shannon diversity of bacterial communities in larvae and pollen samples for different solitary bee species based on revealed bacterial OTUs per nest chamber. Shannon bacterial diversity was consistently higher for pollen samples (gray box plots) in comparison with larvae (black box plots). Right: Shannon diversity of pollen species in pollen provisions for different solitary bee species

Shannon bacterial diversity was higher for pollen samples (gray box plots in Figure 3) compared to larvae (black box plots in Figure 3). The Wilcoxon test between all larvae and pollen samples returned statistically significant results for Shannon values (*p* < .001***). Furthermore, we conducted pairwise Spearman's rank correlations to investigate associations between bacterial diversity for larvae, bacterial diversity for pollen, and pollen‐type diversity. Spearman's correlations for Shannon index values between bacterial communities of larvae and pollen were significant (*ρ* = 0.38, *p* < .001***). In addition, the number of identified pollen species in each sample was significantly correlated with the respective Shannon bacterial diversity in pollen (*ρ* = 0.24, *p* < .05*). Bacterial OTU Shannon diversity values in larvae were not correlated with the respective pollen species number (*ρ* = −0.06, *p* > .05).

### 3.5 Correlation of bacterial and pollen composition

We were able to detect statistically significant Mantel correlations of pollen composition with pollen bacterial communities and also with larval bacterial communities in the whole dataset (Table 1). When we conducted the same tests within each host bee species, we detected significant correlations between pollen species and pollen bacterial taxa for *H. truncorum* (*n* = 35), *M. rotundata* (*n* = 20), and *M. versicolor* (*n* = 4) (Table 1).

**Table 1:** Mantel correlations between Bray–Curtis distance matrices based on presence/absence data for plant species and bacterial OTUs in pollen, plant species, and bacterial OTUs in larvae, as well as for bacteria OTUs in pollen and larvae. *n* stands for the number of nest chambers, and Mantel statistic is indicated with the letter *r*

	Plant species × pollen bacteria	Plant species × larval bacteria	Pollen bacteria × larval bacteria
Whole dataset (*n* = 100)	***r* = .32, *p* < .001** ***	***r* = .18, *p* < .001** ***	***r* = .23, *p* < .001** ***
*H. truncorum* (*n* = 35)	***r* = .18, *p* < .05** *	*r* = −.01, *p* = .52	***r* = .52, *p* < .001** ***
*M. ligniseca* (*n* = 8)	*r* = .03, *p* = .38	*r *= −.02, *p* = .49	*r *= −.13, *p* = .76
*M. rotundata* (*n* = 20)	***r* = .31, *p* < .001** ***	*r* = .15, *p* = .06	***r* = .30, *p* < .01** **
*M. versicolor* (*n* = 4)	***r* = .83, *p* < .05** *	*r* = .18, *p* = .33	*r* = .13, *p* = .33
*O. bicornis* (*n* = 21)	*r* = .17, *p* = .08	*r *= −.14, *p* = .15	*r* = .13, *p* = .17
*O. caerulescens* (*n* = 8)	*r *= −.13, *p* = .70	*r* = .02, *p* = .47	*r *= −.15, *p* = .65
*O. leaiana* (*n* = 4)	*r *= −.48, *p* = .88	*r* = .23, *p* = .42	*r* = .66, *p* = .08

Note

Significant correlations with *p* <  0.05 are listed in bold, and marked with *for *p* <  0.05, **for *p* <  0.01 and ***for *p* <  0.001

### 3.6 Bacterial and pollen beta diversity

**Figure 4:**
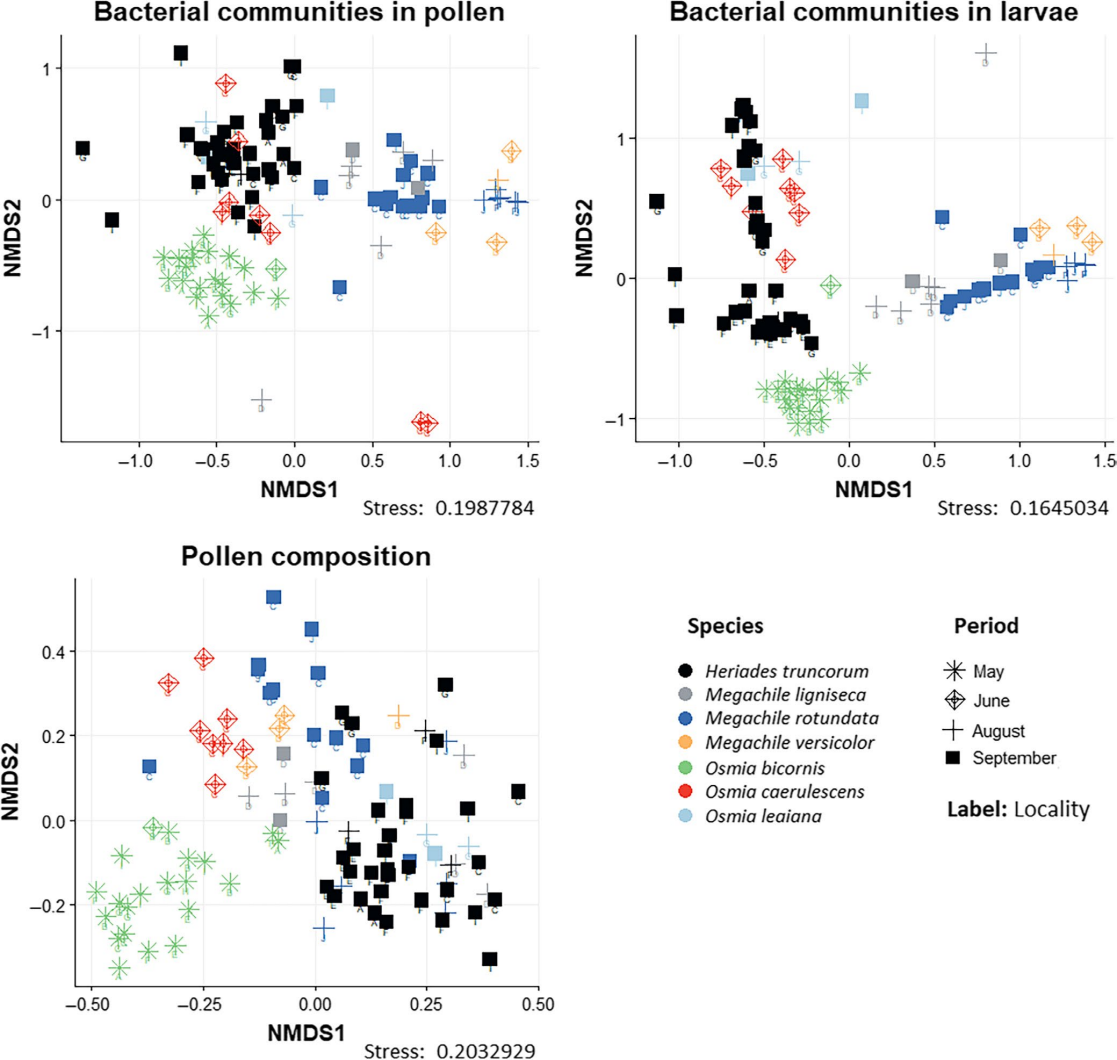
NMDS ordination of all samples. The bacterial community ordinations are based on Bray–Curtis distances of bacterial OTUs, and the pollen composition ordination is based on presence/absence data of plant species. Sample points are colored according to host bee species and shaped according to period of sampling. Labels on the ordination points refer to different sampling sites

The ordinations for the bacterial communities and the pollen composition of all samples are shown in Figure 4. Adonis/PERMANOVA results indicated that bee species identity was more significant as a discriminative factor for pollen composition in our dataset (*R* ^2^ = 0.33, *p* < .001***), comparing to the sampling site (*R* ^2^ = 0.18, *p* < .001***) and sampling period (*R* ^2^ = 0.07, *p* < .001***). However, multivariate dispersions among groups were not homogenous for species and sampling site (betadisper *p* < .01**), lessening the explanatory power of the Adonis test. Also, the sampling location had explanatory power on the shaping of the pollen bacterial communities only for *O. bicornis* (*R* ^2^ = 0.40, *p* < .01**; betadisper *p* < .05*), while it could not explain the structure of the larval bacterial communities for any of the species (all *p* > .05). Sampling period could explain a percentage of the pollen and the larval bacterial communities' variance only for *M. rotundata* (*R* ^2^ = 0.11, *p* < .001***; betadisper *p* > .05 and *R* ^2^ = 0.12, *p* < .001***; betadisper *p* > .05, respectively).

### 3.7 Assignment of bacterial communities to host bee species and pollen composition

**Figure 5:**
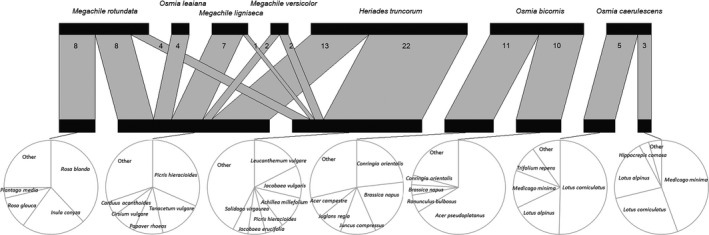
Pollen samples were classified into seven clusters according to their composition in plant species. Pie charts demonstrate the indicator pollen species for each cluster, and the bipartite network shows the number of samples from each bee species assigned to each cluster. The clusters are treated as different whole mixed diets for the respective larvae

We further tested with a cluster analysis whether different dietary habits within and between bee species are associated also with differences in the respective bacterial microbiota. Pollen provisions from all samples were divided into seven clusters according to their composition in plant species. These clusters were treated as different whole mixed diets for individual larvae to which they were assigned. The ecological trait analysis showed that only *O. bicornis* foraged for trees, while all the other bee species visited subshrubs or forbs (Figure 5).

We tested the efficiency of the pollen clusters and of the host bee species identity to predict the respective pollen bacterial communities with random forests (Figure 5). Random forest analysis assigned 77% of all bacterial pollen communities to host species and 70% to pollen composition, correctly. Within each bee species, the assignment of all pollen bacterial communities to pollen composition clusters returned low error rates (0% for *M. ligniseca*, *M. versicolor*, and *O. caerulescens*; 10% for *M. rotundata*; 11.43% for *H. truncorum*; and 14.29% for *O. bicornis*, Appendix S2). For bacterial communities in larvae, the correct assignments were 89% to host species and 58% to pollen composition cluster.

## 4 DISCUSSION

In the present study, we investigated the association between the pollen composition and the nest bacterial microbiota for seven megachilid bee species by comparisons of solitary bee nests both between bee species and within the same bee species. Although a degree of the discovered microbiota variation between different bee species could be attributed to the bee species traits and the possible ability of the mother bee to actively transfer bacteria to her eggs, we showed that the bacterial diversity and composition of the nest is also related to the plant diversity and composition of the provided pollen. Furthermore, associations between specific plants and certain bacterial taxa suggest the importance of possible conserved plant–microbe relationships for the understanding of the bee–plant–microbe triangle (McFrederick et al., 2017; Voulgari‐Kokota, McFrederick, et al., 2019).

### 4.1 Bacterial composition in the nest environment

The investigation of the solitary bee nest bacterial microbiota revealed diverse bacterial communities. Additionally, we found bacterial taxa which were prevalent only in the pollen provisions of some bee species, while they were absent from others. This is the case with *Lactobacillus* spp. in particular; the genus is closely connected with the pollen provisions as well as with the larvae of *Megachile* bees (Figure 1). At the same time, *Lactobacillus* spp. either did not exist (*O. bicornis* and *O. leaiana* samples) or it occurred in small abundances (*H. truncorum* and *O. caerulescens*) in the rest of our samples (Figure 1). The genus is also well known for the social bee larva microbiome (Kwong & Moran, 2016) and the honey beehive environment (Corby‐Harris, Maes, & Anderson, 2014). The most abundant *Lactobacillus* phylotype was taxonomically assigned as *L. micheneri*, a species that was also isolated from wild bee guts and flowers in the United States and quantified in high densities in adults, larvae, and pollen, and lower densities in flowers (McFrederick et al., 2017, 2018). The congruity of these results indicates the existence of a conserved association between *Megachile* spp. and specific Lactobacilli at a global scale. This association could contribute to the nutrition and defense of the larvae, resembling the role of Lactobacilli in honey beehives (Vásquez et al., 2012).

Several gammaproteobacteria were found in higher abundances in pollen rather than in larval samples in our dataset. These include bacteria associated with flowering plants such as *Erwinia* (Gnanamanickam, 2006; Junker & Keller, 2015; Junker et al., 2011), *Pseudomonas* (Garrity, Bell, & Lilburn, 2010), and *Acinetobacter* (Álvarez‐Pérez, Lievens, Jacquemyn, & Herrera, 2013). *Erwinia* has also been reported from wild nesting bee microbiota surveys (McFrederick & Rehan, 2016; Voulgari‐Kokota, Grimmer, et al., 2019). The different levels of typical floral bacteria in pollen and larvae indicate that there is a filter for passive bacterial transmission although larvae developed attached on the pollen provisions, and their microbiota could be a subset of the respective pollen microbiota. Finally, the genus *Sodalis*, which is highly abundant in *M. versicolor*, was recently reported as symbiotic in the eusocial form of several Halictidae (Rubin, Sanders, Turner, Pierce, & Kocher, 2018).

### 4.2 Pollen composition in nest provisions

Our dataset included both oligolectic bee species (*H. truncorum*, *O. caerulescens*, *O. leaiana*) and polylectic generalists (Westrich, 2018). The interaction network illustrating plant–pollinator associations (Figure 1) shows several plant species, with pollen mainly present in the nests of a single bee species. Also, only *O. bicornis* had collected pollen from trees, whereas most other species foraged mainly on herbs and shrubs, indicating the importance of bee diversity for the efficient pollination of a wide variety of plants. Pollen metabarcoding allowed us to discover which plant species consisted the pollen provisions in our nests without the need of palynological observations (Bell et al., 2016; Keller et al., 2015; Richardson et al., 2019; Sickel et al., 2015). Yet, to avoid potential problems with abundance estimations in pollen metabarcoding (Bell et al., 2016; Richardson et al., 2019), we in this study concentrate on the presence/absence‐based analyses.

On the bee side, if plants act as reservoirs or transfer hubs for bacteria (Keller et al., 2018; McFrederick et al., 2017), then specialized bee–plant interactions in a landscape could secure specialized bee–bacteria relationships. Thus, plant availability and specialized interactions with plants would be significant not only for the nutrition of the bees, but also for the maintenance of their nest microbiota.

### 4.3 Co‐occurrence of plant species with bacterial OTUs in the pollen

Co‐occurrence analysis enabled us to look into bacterial taxa from pollen, which might be associated with specific plants. On the one hand, we expected to see bacterial taxa which are commonly associated with plants (Gnanamanickam, 2006; Junker & Keller, 2015; Junker et al., 2011) to be part of co‐occurrence relationships with various plant species. The genus *Pseudomonas*, for instance, was associated with most plant species as randomly distributed (Veech, 2013), while the genus *Erwinia* co‐occurred particularly with plants from different families such as Asteraceae, Apiaceae, Juncaceae, and Brassicaceae (Figure 2).

On the other hand, co‐occurrence analysis can help us focus on bacterial taxa which are likely to adopt key functions for the larval health. More specifically, *Lactobacilli* could be acquired from several *Asteraceae* plants, with which they have a positive co‐occurrence relationship (Figure 2). *Achillea millefolium* in particular, which was associated with *Lactobacillus*, was visited by all three *M. rotundata*, *H. truncorum*, and *O. leaiana*. *O. bicornis* bees, on the other hand, did not feed on Asteraceae, and the existence of Lactobacilli in their provisions and larvae was very low. Since the co‐occurrence analysis was based on bee‐collected pollen and not on pollen from the visited flowers, it does not prove transfer of the associated bacteria in the nests through foraging. However, it provides information for possible associations, which can be the subject of further investigation on the floral microbiota of bee‐visited plants.

### 4.4 Bacterial and plant diversity in megachilid bee nests

Introduced pollen in solitary bee nests could be a major bridge for bacterial colonization inside the nest chambers (McFrederick et al., 2017). Thus, diversity in plant sources could also contribute to its bacterial diversity. Our results showed a weak, yet statistically significant correlation between bacterial alpha diversity and the number of pollen species in the pollen provisions. Larvae develop in close proximity with the provided pollen provision, and the diversity of their bacterial communities was also significantly correlated with the respective ones of the pollen provisions. Nevertheless, the pollen bacterial communities were consistently more diverse than the ones in larvae (Figure 3). This difference in diversity could indicate that the larvae can inhibit growth of environmentally introduced bacteria, as previously proposed (Voulgari‐Kokota, Grimmer, et al., 2019); the mechanisms behind that, however, are yet unknown.

### 4.5 Association between pollen composition and bacterial microbiota

Our dataset consisted of seven solitary bee species sampled across numerous sites of a geographical region. Therefore, their revealed foraging patterns could be the result of spatial and temporal factors. The shaping of the solitary bee nest microbiota could be influenced by such factors as well, and it could also show variance depending on the age of the nest. Before focusing on the relation of the collected pollen to the nest bacterial microbiota, we examined the effect of the sampling location and the sampling date on the pollen and bacterial composition of all samples. Location and sampling period did have an effect on foraging patterns; it was, however, with less explanatory power than the effect of the bee species identity. At the same time, these factors were not significant in explaining pollen microbiota variation within bee species in our dataset.

In general, the pollen composition was significantly correlated with the bacterial community in pollen, as well as with the bacterial community in larvae through our whole dataset (Table 1). This result, however, does not necessarily mean that the pollen drives the bacterial covariance, since the ability of each bee species to inoculate the collected pollen with specific bacteria is largely unexplored. To focus deeper on the pollen's association with the nest microbiota, we further concentrated on intraspecific associations. Correlations of pollen composition with bacterial communities within each host bee species returned significant results for some bee species, but not for all. It has been previously proposed that the influence of the pollen composition on the pollen provision microbiota can be masked when examined at a small scale (for instance, when interactions within a bee species or within few samples are investigated), while the same influence can be made apparent, when different bee species with distinctive foraging preferences and larger sample numbers are compared (McFrederick et al., 2017). In our case, within‐species correlations between pollen and bacterial composition in pollen were significant for the two of the most represented species in our dataset (*H. truncorum* with *n* = 35 and *M. rotundata* with *n* = 20). Furthermore, we observed correct random forest assignment of pollen bacterial communities to the respective pollen provisions, when the pollen composition of each species was classified into groups representing different composite diets. Successful assignment of pollen bacterial communities to each composite pollen diet within each host species showed that the nest microbiota of a species reflected the pollen provided to the larvae (Figure 5, Appendix S2).

## 5 CONCLUSIONS

**Figure 6:**
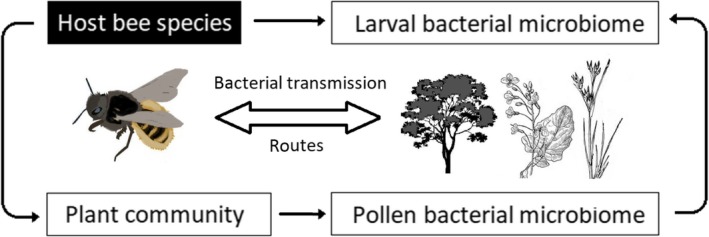
The parallel investigation of the nest bacterial communities and of the pollen composition suggests that floral resource exploitation plays a role in bacterial acquisition and host–microbe interactions

Specialized plant–bee relationships could lead to specialized bee–microbe interactions, making passive transmission through imported pollen an important driver of the natural bee nest microbiota. In the present study, we were able to reveal distinctive foraging patterns for seven bee host species, as well as to describe the bacterial communities of their nests. The parallel investigation of the nest bacterial communities and of the pollen composition allowed us to propose host–microbe interactions, which might be secured through floral resource exploitation (Figure 6). Pollen alpha diversity was correlated with the respective bacterial diversity in the pollen provisions. Also, the pollen community structure was significantly correlated with that of the pollen bacterial communities for the whole dataset and as well within some bee species. Moreover, pollen composition was a successful predictor for the bacterial microbiota at an intraspecific level. Future studies should include more bee species, larger sample sizes, and investigation of the floral microbiota of actual bee‐visited plants for a direct comparison with the acquired larval bacterial community in the nests. Such dedicated experiments will increase our understanding of the causality behind the observed covariance between pollen composition and bacterial microbiota in the nests of solitary bees and to interpret its importance for their health and fitness.

## CONFLICT OF INTEREST

None declared.

## AUTHOR CONTRIBUTIONS

AK, AV‐K, and IS‐D designed the study. AV‐K and GG performed laboratory and fieldwork. AV‐K, MJA, and AK analyzed data. AV‐K drafted the manuscript, and all authors contributed to the final version.

## Data Availability

Raw sequencing data for 16S rDNA and ITS2 rDNA have been deposited at the Sequence Read Archive (SRA) at the European Nucleotide Archive (https://www.ebi.ac.uk/ena) and are publicly accessible under project numbers PRJEB27223 (Voulgari‐Kokota, McFrederick, et al., 2019) and PRJEB31610.
