# Supplementary material for: Linking pollen foraging of megachilid bees to their nest bacterial microbiota[image]
Source: Ecol Evol. 2019 Sep 2;9(18):10788–800. doi: 10.1002/ece3.5599 (PMC6787775; doi:10.1002/ece3.5599)
Supplement: Supplementary file 2 [file ECE3-9-10788-s002.docx]

**Supplementary table .** Random forest analysis shows the adequacy of the host species identity and the pollen composition as indicative factors predicting the pollen bacterial communities. Confusion matrices show the number of correctly assigned communities to each of the two factors, the proportional class error for each category and the total OOB estimate of the error rate.

| Assignment of pollen bacterial communities to bee host species | | | | | | | | | | | | | | | |
| --- | --- | --- | --- | --- | --- | --- | --- | --- | --- | --- | --- | --- | --- | --- | --- |
| OOB estimate of error rate: 23% | | | | | | | | | | | | | | | |
| Confusion matrix: | | | | | | | | | | | |  | |  |  |
|  | | *H. truncorum* | | *M. ligniseca* | | *M. rotundata* | | *M. versicolor* | | *O. bicornis* | | *O. caerulescens* | | *O. leaiana* | class error |
| *H. truncorum* | | 34 | | 0 | | 0 | | 0 | | 0 | | 1 | | 0 | 0.02857143 |
| *M. ligniseca* | | 1 | | 2 | | 4 | | 0 | | 0 | | 1 | | 0 | 0.75 |
| *M. rotundata* | | 1 | | 1 | | 18 | | 0 | | 0 | | 0 | | 0 | 0.1 |
| *M. versicolor* | | 0 | | 0 | | 4 | | 0 | | 0 | | 0 | | 0 | 1 |
| *O. bicornis* | | 0 | | 0 | | 0 | | 0 | | 21 | | 0 | | 0 | 0 |
| *O. caerulescens* | | 4 | | 0 | | 0 | | 0 | | 2 | | 2 | | 0 | 0.75 |
| *O. leaiana* | | 4 | | 0 | | 0 | | 0 | | 0 | | 0 | | 0 | 1 |
| Assignment of pollen bacterial communities to pollen composition cluster | | | | | | | | | | | | | | | |
| OOB estimate of error rate: 30% | | | | | | | | | | | | | | | |
| Confusion matrix: | | | | | | | | | | | | | | | |
|  | | Cluster 1 | | Cluster 2 | | Cluster 3 | | Cluster 4 | | Cluster 5 | | Cluster 6 | | Cluster 7 | class error |
| Cluster 1 | | 8 | | 0 | | 0 | | 0 | | 0 | | 0 | | 0 | 0 |
| Cluster 2 | | 0 | | 9 | | 0 | | 0 | | 0 | | 2 | | 0 | 0.1818182 |
| Cluster 3 | | 0 | | 0 | | 0 | | 2 | | 1 | | 0 | | 0 | 1 |
| Cluster 4 | | 0 | | 0 | | 0 | | 26 | | 8 | | 0 | | 0 | 0.2352941 |
| Cluster 5 | | 1 | | 0 | | 0 | | 8 | | 20 | | 0 | | 0 | 0.3103448 |
| Cluster 6 | | 0 | | 3 | | 0 | | 0 | | 0 | | 7 | | 0 | 0.3 |
| Cluster 7 | | 0 | | 1 | | 0 | | 1 | | 2 | | 1 | | 0 | 1 |
| Assignment of pollen bacterial communities to pollen composition cluster for: | | | | | | | | | | | | | | | |
| - *H. truncorum* | | | | | | |  | | | | - *M. ligniseca* | | | | |
| OOB estimate of error rate: 11.43% | | | | | | |  | | | | OOB estimate of error rate: 0% | | | | |
| Confusion matrix: | | | | | | |  | | | | Confusion matrix: | | | | |
|  | Cluster 4 | | Cluster 5 | | class error | |  | | | |  | | Cluster 4 | Cluster 5 | class error |
| Cluster 4 | 10 | | 3 | | 0.23076923 | |  | | | | Cluster 4 | | 7 | 0 | 0 |
| Cluster 5 | 1 | | 21 | | 0.04545455 | |  | | | | Cluster 5 | | 0 | 0 | NaN |
| - *M. rotundata* | | | | | | | | | | | - *M.versicolor* | | | | |
| OOB estimate of error rate: 10% | | | | | | | | | | | OOB estimate of error rate: 0% | | | | |
| Confusion matrix: | | | | | | | | | | | Confusion matrix: | | | | |
|  | Cluster 1 | | Cluster 4 | | Cluster 5 | | class error | |  | |  | | Cluster 4 | Cluster 5 | class error |
| Cluster 1 | 8 | | 0 | | 0 | | 0 | |  | | Cluster 4 | | 2 | 0 | 0 |
| Cluster 4 | 0 | | 8 | | 0 | | 0 | |  | | Cluster 5 | | 0 | 2 | 0 |
| Cluster 5 | 1 | | 1 | | 2 | | 0.5 | |  | |  | |  |  |  |
| - *O. bicornis* | | | | | | | | | | | - *O. caerulescens* | | | | |
| OOB estimate of error rate: 14.29% | | | | | | | | | | | OOB estimate of error rate: 0% | | | | |
| Confusion matrix: | | | | | | | | | | | Confusion matrix: | | | | |
|  | Cluster 2 | | Cluster 6 | | class error | |  | | | |  | | Cluster 3 | Cluster 7 | class error |
| Cluster 2 | 9 | | 2 | | 0.1818182 | |  | | | | Cluster 3 | | 3 | 0 | 0 |
| Cluster 6 | 1 | | 9 | | 0.1 | |  | | | | Cluster 7 | | 0 | 5 | 0 |
